# Religion and Satisfaction with Life in Polish Seniors: Mediation by Forgiveness and Hope

**DOI:** 10.1007/s10943-024-02070-z

**Published:** 2024-06-04

**Authors:** Elżbieta Rydz, Anna Tychmanowicz, Beata Zarzycka

**Affiliations:** 1 Institute of Psychology, The John Paul II Catholic University of Lublin, Lublin, Poland; 2 Institute of Psychology, The Maria Curie-Skłodowska University in Lublin, Głęboka Street 45, 20-612 Lublin, Poland

**Keywords:** Religiousness, Forgiveness, Hope, Satisfaction with life, Polish seniors

## Abstract

This study examined the mediating role of forgiveness and hope in the relationship between religiousness and satisfaction with life in late adults in Poland. Participants were 237 people (165 females and 72 males) aged between 60 and 92. The mean age of the sample was 68.37 years (*SD* = 6.92), and the most represented religious affiliation was Roman Catholic (98.3%). Satisfaction with life is related to the centrality of religiousness. In the surveyed seniors, hope and tendency to forgive mediated the relationship between the centrality of religiousness and satisfaction with life.

## Introduction

Previous studies have suggested that religiosity can promote life satisfaction (Garssen et al., 2021; Sholihin et al., 2022; Yaden et al., 2022). However, the intensity of these relationships remains a matter of debate (Yaden et al., 2022). The results of the meta-analysis by Sholihin et al. (2022) showed that religiosity is a significant explanatory and determinant of life satisfaction (effect size overall statistical value of 2.85, 95% CI, 1.81–3.82). Similarly, a meta-analysis by Garssen et al. (2021) involving longitudinal studies on religiosity and selected aspects of mental health, including life satisfaction, indicates a significant although small effect size (*r* = 0.10, 95% CI, 0.06, 0.13; *p* = 0.000) between overall religiosity and life satisfaction. In explaining and understanding the relationship between religiosity and life satisfaction, it is essential to consider the indicators or aspects of religiousness under study.

Two dimensions of religiousness may be necessary in the context of quality of life and life satisfaction: (1) religious beliefs, which determine a coherent worldview, interpretation of events, and sense of meaning, and (2) private practices, such as prayer or religious reading, which can increase feelings of security, group membership, perceived control, and help with problem-solving (Sholihin et al., 2022; Ten Kate et al., 2017). Similarly, research by Zotti et al. (2016) indicates that religious affiliation, frequent attendance at religious services, and the belief that religion is essential in one’s life are important for well-being/life satisfaction.

Metanalysis by Yaden et al. (2022) indicated that (1) spirituality, (2) religiosity, (3) attendance at religious services, (4) religious/spiritual practices, and (5) religious/spiritual experiences predicted life satisfaction. The results revealed an effect size of *r* = 0.18 (95% CI, 0.16, 0.19; *p* < 0.01) for overall religiosity and individual aspects ranging from r = 0.11 (95% CI, 0.09, 0.13; *p* < 0.01) for religious attendance to *r* = 0.30 (95% CI, 0.25, 0.35; *p* < 0.01 for spirituality. The results of these meta-analyses show that each of the dimensions of religiosity examined was significantly and positively associated with life satisfaction, but the association was stronger in less developed countries (*b* = −0.09, *p* = 0.02), more religious countries (*b* = 0.002, *p* = 0.01), and with higher age of respondents (*b* = 0.002, *p* = 0.002) (Yaden et al., 2022). Age is an important variable that can determine religiosity, life satisfaction, and the connections between the two (Bergan & McConatha, 2001; Yaden et al., 2022).

Numerous studies have supported that religiousness has an essential impact on life satisfaction in late adulthood (Bergan & McConatha, 2001; Lee, 2011; Van Ness & Larson, 2002), but less is known about the patterns of this association, including the potential mediators. By determining the factors mediating the relationship between religiousness and well-being, one can establish the determinants of seniors’ life satisfaction, which can foster positive aging.

In investigating successful aging, researchers focus on measuring individuals’ well-being, an important component of which is satisfaction with life (Diener & Biswas-Diener, 2008). Previous studies suggest that life satisfaction is significantly related to religiosity (Koenig et al., 1988; Krause, 2003; Park, 2005; Platsidou, 2012). Faith can play a role in seniors’ lives because as life’s perspective becomes shorter, questions about what comes next, the prospect of life after death, come to the fore (Dillon & Wink, 2007; Koenig, 2012). The desire to provide deeper meaning to the inevitable losses that old age brings is also intensified (Hood et al., 2019; McFadden, 2005). Moreover, religion offers opportunities to build social ties in a community of believers (Hood et al., 2019), and it may also present its adherents with principles for (re)building positive interpersonal relationships based on benevolence and forgiveness (Pargament & Rye, 1998). Basic hope, defined as the belief in the world’s meaningfulness, predictability, and generosity, is positively associated with life satisfaction and can also be related to religiousness (Krok, 2013).

The literature offers theoretical models to help explain the relationship between religiousness and well-being. An example is the spiritual framework of coping (SFC) by Gall et al. (2005). The SFC refers to the concept of spiritual/religious meaning-making and draws on the transactional model of coping with stressful events (Lazarus & Folkman, 1984). In the SFC model, an individual’s well-being under challenging circumstances results from the complex processes of making spiritual/religious meaning in interacting conditions. Religiousness precedes well-being, while personal factors, e.g., hope, problem-solving, and forgiveness, can mediate the relationship between religiousness and well-being.

Park and Slattery (2013) developed a bidirectional model of the mediational pathways via which different dimensions of religiosity can promote or hinder mental illness, mental health, well-being, and thriving and vice versa. Religiosity, through various paths, can improve mental health, for example, via enhancing social support, providing guidelines for living, encouraging forgiveness, creating a positive relationship with God, strengthening religious coping resources, etc. The dimensions of religiosity that can be harmful to mental health include negative religious attributions, negative social interactions, and negative affect. Park and Slattery (2013) considered forgiveness as one of the mediators of the relationships between religiousness and mental health/well-being. The search for links between religiosity and indicators of well-being and the mechanisms that explain these relationships is still an open research perspective.

The current study aims to analyze the mediating role of forgiveness and hope in the relationship between religiosity and life satisfaction in seniors. This study extends existing knowledge about the role of religiosity, forgiveness, and hope in health and well-being, specifically for people as they age.

### Religion and Satisfaction with Life in Late Adulthood

Satisfaction with life is defined as global contentment with life as a whole (Diener & Biswas-Diener, 2008). It includes a cognitive and affective evaluation of one’s life based on comparing one’s current life with expectations (Diener et al., 2003; Prasoon & Chaturvedi, 2016). Many studies so far have shown that people with higher levels of religiousness/spirituality experience higher satisfaction with life (Krause, 2003; Park, 2005; Platsidou, 2012) and that the relationship is stronger in older than in younger adults (Bergan & McConatha, 2001). This relationship is explained, among others, by the fact that religion offers a sense of belonging and participation in a community, which is a source of support in difficult life situations (Bergan & McConatha, 2001; Platsidou, 2012).

The authors of some of these studies suggest that life satisfaction may also be fostered by prayer and establishing a relationship with God, which can give people a sense of support, add meaningfulness to life events, or enhance people’s sense of worth and self-efficacy (Bergan & McConatha, 2001; Kate et al., 2017). Some studies show greater complexity of the relationship between prayer and life satisfaction, considering different types of worship. For example, Masters and Spielmans’ (2007) meta-analysis of distant intercessory prayer shows that respondents do not derive significant benefits from it (mean effect size was *g* = 0.082; *p* = 0.26). Similarly, research by Newman and colleagues (2023) considering four types of prayer (adoration, confession, thanksgiving, and supplication) shows a significant association of prayer with life satisfaction only for thanksgiving (effect size *r* = 0.26, *p* < 0.001) and adoration (effect size *r* = 0.24, *p* < 0.001).

It has also been reported that the relationship between religiousness and satisfaction with life is explained by religious coping (Pargament & Rye, 1998; Pargament et al., 1998) and religious meaning (Krause, 2003). By strengthening the sense of purpose in life, religion encourages health-protecting and health-promoting practices (Homan & Boyatzis, 2010) while discouraging undesirable behaviors (Krause, 2003), which can enhance the well-being of older adults.

### Forgiveness and Satisfaction with Life in Late Adulthood

Psychology defines forgiveness as a complex process involving emotional, cognitive, and behavioral changes. In social psychology, researchers describe forgiveness as a motivational construct, which is, at the same time, a pro-social change in the motivational system in response to a hurtful interpersonal offense (McCullough & Witvliet, 2002). Forgiveness stimulates the motivation to replace negative emotions with positive ones by breaking the vicious cycle of avoidance–revenge and is unrelated to accepting, forgetting, or denying the wrongdoing (McCullough & Witvliet, 2002). Forgiveness can also be considered a coping strategy that reduces physical, psychological, and interpersonal stress (Worthington & Scherer, 2004). Many researchers believe forgiveness is rooted in religiousness since major world religions encourage and promote forgiveness (Hantman & Cohen, 2010), including Judaism, Christianity, and Islam (Zarzycka, 2019).

The concept of forgiveness may refer to others (interpersonal forgiveness), to oneself (self-forgiveness) (Davis et al., 2015), and to God (feeling forgiven by God) (Toussaint et al., 2001). This study focuses on interpersonal forgiveness. Some studies indicated individual differences in the propensity to forgive (Brown & Philips, 2005), differentiating between attitudes and tendency to forgive (Brown, 2003; Brown & Philips, 2005).

Research confirmed the positive relationships between forgiveness and satisfaction with life and subjective well-being in adults. A meta-analysis by Rasmussen and colleagues (2019) indicated that higher forgiveness is associated with higher subjective well-being (effect size *r* = 0.32, 95% CI, 0.26, 0.39, *p* < 0.001). Interpersonal forgiveness was found to be more closely related to life satisfaction and well-being in older adults than in young adults (Tian & Wang, 2021; Toussaint et al., 2001). Cognitive changes humans undergo over their lifetime, including moving away from an egocentric perspective towards empathetic acceptance and appreciation of other people’s viewpoints (Hantman & Cohen, 2010), can make forgiving others easier as people age. This is important because memories of past transgressions accumulated throughout life can give rise to anger, guilt, or insult in old age (Hantman & Cohen, 2010). Forgiveness can be an effective strategy for minimizing the impact of negative experiences and thus enhancing life satisfaction in late adulthood (Allemand et al., 2008; Tian & Wang, 2021).

### Hope and Satisfaction with Life in Late Adulthood

Hope can be defined as (1) the expectation of a sensible, orderly, and benevolent world, both for present and future situations (basic hope) (Erikson, 1982; Trzebiński & Zięba, 2004), and (2) an anticipation that one will achieve success and the belief that success is related to one’s competences (Snyder, 2002). The present study focuses on basic hope defined as "a fundamental belief in two features of the world (a) its order and meaning, and (b) its positiveness” (Trzebiński & Zięba, 2004, p. 174). Basic hope is positively associated with optimism, positive mood, a lower level of anxiety, and life satisfaction (Garoon et al., 2016; Klein, 2016), as well as in late adulthood (Baumann et al., 2020).

Older adults who display higher levels of hope use more adaptive ways of coping with biological and interpersonal losses (Nosraty et al., 2015). The level of hope may be related to their religious affiliation and how deeply religious they are (Van Hook, 2016). Religiousness is a potential source of hope, related, among other things, to people’s beliefs about life after death, which can provide reassurance and comfort, even though they may differ across religious traditions/denominations (e.g., Christian vs. Hindu) (Van Hook, 2016).

### Possible Mediator: Forgiveness and Hope

We propose forgiveness and hope as possible mechanisms explaining the relationship between religiosity and life satisfaction (Abu-Raiya & Ayten, 2020). Understanding these relationships may be particularly important for elucidating the determinants of well-being in older adults (Lawler-Row, 2010; Lawler-Row & Elliott, 2009).

In their study of the associations between religiousness, forgiveness, and health, Lawler-Row and Elliott (2009) found that feeling forgiven by God mediated the relationships between religiousness and the seniors’ sense of successful aging. Forgiveness mediated the relationship between closeness to God and improved well-being (Torges et al., 2013). Similarly, the mediating role of interpersonal forgiveness in the relationship between religious involvement and life satisfaction was demonstrated in a sample of Muslim participants (Abu-Raiya & Ayten, 2020). However, forgiveness did not significantly mediate the relationship between religious beliefs and life satisfaction (Abu-Raiya & Ayten, 2020).

In the elderly, the mediating role of forgiveness in the relationship between religiosity and well-being may be either full or partial. Research by Lowler-Row (2010) showed that feeling forgiven by God fully mediated the relationship between frequency of attendance, frequency of prayer, and belief in a watchful God with successful aging. Self-forgiveness and interpersonal forgiveness partially mediated the relationship between religiosity and well-being in late adulthood.

We also proposed hope as a mediator of the relationship between religiosity and life satisfaction, which is hope. Previous research indicated that in late adulthood, religiosity is positively associated with hope, and the latter positively correlates with life satisfaction (Pahlevan Sharif et al., 2021). When people face the physical deterioration and loss inherent in aging, religiosity can be a source of hope. That hope can help seniors deal with difficulties, illnesses, and bereavement, thus fostering their well-being (Trede, 2006). The mediating role of hope in the relationship between religiosity and mental health in people at the end of life has also been suggested in the meta-analysis conducted by Van Ness and Larson (2002). All these data have led us to the expectation that forgiveness and hope mediate the relationship between religiosity and life satisfaction among seniors.

In the context of the findings reported by other authors, we formulated two research hypotheses:

#### H1

Forgiveness mediates the relationship between the centrality of religiosity and satisfaction with life in late adulthood.

#### H2

Hope mediates the relationship between the centrality of religiosity and satisfaction with life in late adulthood.

## Method

### Participants

Participants were 237 people (165 females and 72 males) aged between 60 and 92. The mean age of the sample was 68.37 years (SD = 6.92), and the most represented religious affiliation was Roman Catholic (98.3%). The proportion between the number of male and female participants corresponds with the current demographic trend for this age group in Poland. Table 1 shows baseline demographic characteristics for all participants. Respondents answered questions about religious activity, attending religious services, and private prayer. Of those surveyed, 171 people participated in religious services at least once weekly (including via radio or television). The others gave the following answers: every two weeks (*n* = 12), once a month (*n* = 11), several times a year (*n* = 37) and never (*n* = 6). Personal prayer is prayed at least once daily by 94 people and several times weekly by 89. The remaining people gave the following answers: at least once a week (*n* = 40), several times a year (*n* = 11) and never (*n* = 3).

**Table 1:** Demographic Characteristics of Participants

Variable	N	%
*Sex*		
Male	72	30.4
Female	165	69.6
*Marital status*		
Unmarried	11	4.6
Married	160	67.5
Divorced	8	3.4
Widow/er	58	25.5
*Having children*		
Yes	230	97.0
No	7	3.0
*Place of residence*		
Village	96	40.5
Town (< 200 000)	80	33.8
Big city (> 200 000)	61	25.7
*Religious affiliation*		
Roman Catholicism	233	98.3
Greek Catholicism	1	0.4
Atheism	3	1.3

### Procedure

The study received ethical approval from the Ethics Committee for the John Paul II Catholic University of Lublin (KEBN_24/2020). Participants were recruited at social care homes, seniors’ clubs, and other institutions for seniors, with the approval of the heads of these organizations. The inclusion criteria were age and self-reported good health status. Persons with poor health status (e.g., with psychiatric or neurological disorders) did not take part in the study. The inclusion criteria were consulted with medical caregivers in the institutions where the survey was conducted. Participants were surveyed across Poland, but most came from eastern Poland. The eastern regions of Poland, which were most highly represented in this study, are characterized by higher levels of religious affiliation and self-declarations of faith (Buzalka, 2008; Himniak, 2020; Zaręba, 2008) compared to the western part of the country, which was less numerously represented in the survey.

A team of trained investigators conducted the survey from January to June 2018. We used Polish adaptations of the questionnaires, and research was conducted using the pencil-and-paper method. Participation in the study was voluntary and anonymous. Informed consent was obtained from all participants. Researchers handed out survey sets, assisting with completion if required (e.g., needing to read or clarify text in the questionnaire). In addition, by picking up the completed questionnaires, the researchers verified deficiencies and asked for completion if necessary. Participants were informed about the aggregate processing of the data obtained, and feedback on the group analysis results was provided.

### Measures

Measures were scored by averaging across items. Descriptive statistics (means, standard deviations) are available in Table 2.

**Table 2:** Means, Standard Deviations, and Correlations Between Centrality of Religion, Tendency to Forgive, Attitude toward Forgiveness, Hope, and Satisfaction with Life (N = 237)

Variable	Centrality	Tendency	Attitude	Hope	Satisfaction
Centrality					
Tendency	.17**				
Attitude	.29***	.40***			
Hope	.27**	.18*	.24**		
Satisfaction	.19**	.16*	.09	.25**	
*M*	3.82	3.64	4.49	3.82	4.14
*SD*	0.83	1.21	1.00	0.51	1.16
*Alpha*	.94	.73	.71	.67	.82

*p < .05 **p < .01 ***p < .001

#### Religiousness

We used the 15-item Centrality of Religiosity Scale (CRS) by Huber (Huber, 2003; Huber & Huber, 2012) to measure religiousness. This instrument is grounded theoretically in Glock and Stark’s (1965) concept of religiosity as a multidimensional structure and Allport’s (Allport & Ross, 1967) view of religiosity as having a motivational function. Huber (2003) adopted a structural approach to religiosity and described its motivational functions in terms of personal constructs, referring to Kelly’s conception (1955).

CRS comprises five dimensions of centrality: intellect, ideology, private practice, public practice, and religious experience. The response options for intellect, ideology, and religious experience were from 1 (not at all/never) to 5 (very much so/very often). For private and public practice, as they are undertaken regularly in most religious traditions and are easily accessible in frequency format, objective frequencies were asked. The response options for private practice were from 1 (never) to 9 (several times a day), and for public practice, they were from 1 (never) to 8 (more than once a week). The objective frequencies were recoded into five levels of subjective frequencies. In this study, we used the total score, Centrality (e.g., “To what extent do you believe that God or something divine exists?”), the sum of the subscale scores. The Cronbach’s alpha value for Centrality in this sample was 0.94.

#### Satisfaction with Life

We used the 5-item Satisfaction with Life Scale (SWLS) by Diener et al. (1985) to measure global cognitive judgments of satisfaction with one’s life (e.g., “In most ways, my life is close to my ideal”). Respondents indicated the extent to which they agreed with each item on a 7-point scale ranging from 1 (*strongly disagree*) to 7 (*strongly agree*). The Cronbach’s alpha value for the SWLS in this study was 0.82.

#### Attitude Toward Forgiveness

We used the 6-item Attitude Toward Forgiveness Scale (ATF) by Brown (2003) to assess individuals’ beliefs regarding forgiveness (forgiving attitude), whether they perceive forgiveness as positive or negative (e.g., “I believe that forgiveness is a moral virtue; it is admirable to be a forgiving person”). The response options were from 1 (*strongly disagree*) to 7 (*strongly agree*). The reliability of the ATF was satisfactory in this sample. The Cronbach’s alpha value for the ATF in the present study was 0.71.

#### Tendency to Forgive

We used the 4-item Tendency to Forgive Scale (TTF) by Brown (2003) to assess the extent to which the participants typically experienced or engaged in forgiveness when others had wronged them. The TTF items describe people’s behaviors in response to a transgression (e.g., “I tend to get over it quickly when someone hurts my feelings; I have a tendency to harbor grudges”). The respondents were asked to assess how each of these behaviors describes their reactions to the offenses experienced so far (Brown & Philips, 2005). The response options were from 0 (*strongly disagree*) to 6 (*strongly agree*). The Cronbach’s alpha value of the TTF in this sample was 0.73.

#### Hope

We used the 12-item Basic Hope Inventory (BHI-12, Trzebiński & Zięba, 2003) to measure the strength of basic hope, understood as beliefs of the basic attributes of the world: order, sense, and kindly attitude. The Basic Hope Questionnaire BHI-12 contains 12 items, and 9 of them are diagnostic (e.g., “There will always be some people to help us in difficult times”). The respondents rate how much they agree with each item on a 5-point scale from 1 (*strongly disagree*) to 5 (*strongly agree*). The Cronbach’s alpha for the total score in this study was 0.67.

### Statistical Analyses

This was a cross-sectional study. To establish whether the key constructs – the centrality of religiosity, hope, tendency to forgive, attitude toward forgiveness, and satisfaction with life – were correlated, we calculated Pearson’s correlations. Next, two mediation analyses were conducted. In both mediation models, the centrality of religiosity was examined as a predictor of satisfaction with life. In the first analysis, hope, whereas in the second analysis, the tendency to forgive and attitude toward forgiveness were investigated as mediators in the relationship between the centrality of religiosity and satisfaction with life. Both mediation analyses were performed using the PROCESS macro (Hayes, 2018). PROCESS utilizes a regression-based path-analytic framework to estimate indirect effects and bias-corrected confidence intervals (CIs) using the bootstrapping method. An indirect effect is considered significant when the CI does not include zero. The analyses were based on 5000 bootstrapping samples. Bootstrapped 95% confidence intervals were presented for all indirect effects. The correlation analysis was performed using SPSS version 25.0. Standardized coefficients are presented.

## Results

The mean scores on the centrality of the CRS, the hope of the BHI, and satisfaction with life of the SWLS were negatively skewed (ranging from −0.19 to −0.99), with a larger number of high values. The mean scores on the tendency to forgive and attitude toward forgiveness scales were positively skewed, 0.27 and 0.12, respectively, with a larger number of low values. The skewness was not strong enough so that it can be ignored. Table 2 shows Pearson’s correlation coefficients between the variables measured in this study. The centrality of religiosity was significantly positively correlated with hope, the tendency to forgive, the attitude toward forgiveness, and satisfaction with life. Hope and tendency to forgive also correlated positively with satisfaction with life. No significant correlations emerged between satisfaction with life and attitude toward forgiveness.

Next, we examined tendency, attitude toward forgiveness, and hope as mediators in the relationship between the centrality of religion and satisfaction with life among people in late adulthood. We conducted a mediation analysis with tendency to forgive and attitude toward forgiveness as parallel mediators in the centrality–satisfaction with life relationship. The indirect effect of centrality of religiosity through tendency to forgive on satisfaction with life was significant (IE = 0.16, [LLCI = 0.001, ULCI = 0.405]). The centrality of religiosity was positively related to the tendency to forgive (*b* = 0.17, *p* = 0.012), which in turn increased satisfaction with life (*b* = 0.13, p = 0.042). The direct effect of the centrality of religiosity on satisfaction with life was also significant (*b* = 0.17, *t* = 2.45, *p* = 0.015). The total effect was also significant (TE = 1.29, *t* = 2.82, *p* = 0.005, [LLCI = 0.385, ULCI = 2.187]. The indirect effect of the centrality of religiosity through attitude toward forgiveness on satisfaction with life was insignificant (IE = 0.01, [LLCI =  − 0.022, ULCI = 0.056]). The direct effect of the centrality of religiosity on satisfaction with life was significant (*b* = 0.17, t = 2.52, *p* = 0.012). The total effect was also significant (TE = 1.29, *t* = 2.83, *p* = 0.005, [LLCI = 0.394, ULCI = 2.193]. Thus, the centrality of religiosity directly predicts satisfaction with life, but not through its effect on attitude toward forgiveness.

**Fig. 1:**
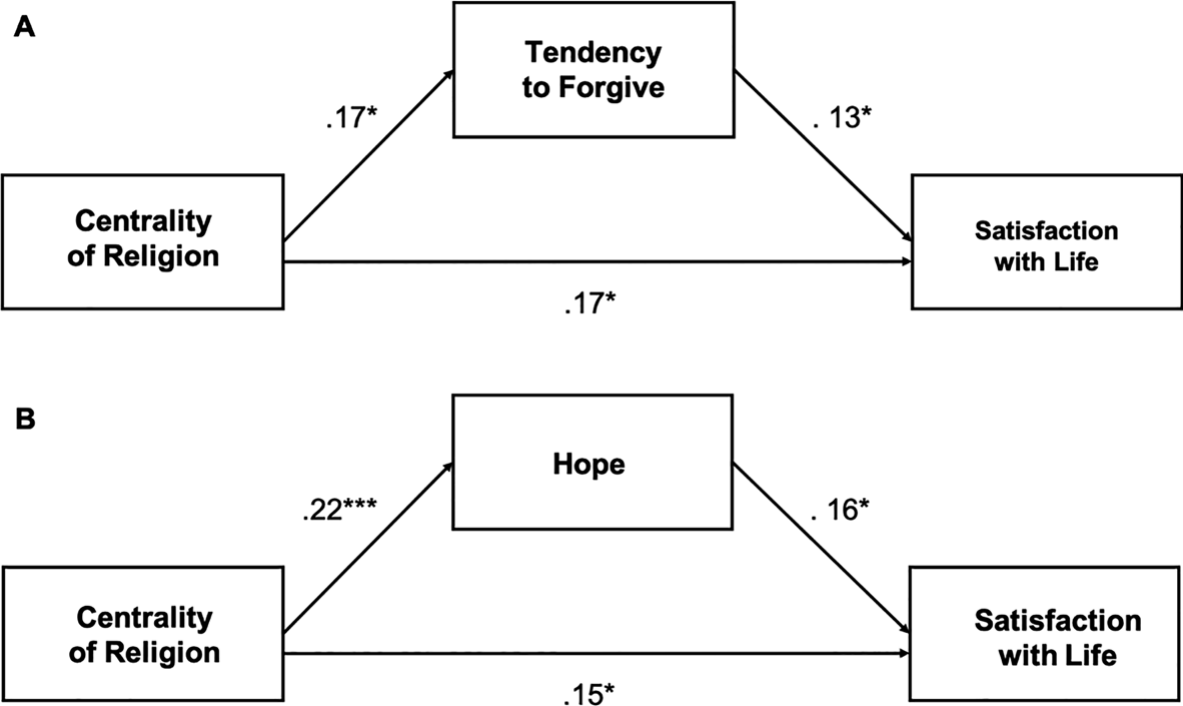
Standardized Regression Coefficients for Relationships Between Centrality of Religiosity and Satisfaction with Life Mediated by (**A**) Tendency to Forgive and (**B**) Hope

Then, we conducted a mediation analysis with hope as a mediator in the centrality–satisfaction with life relationship. The indirect effect of the centrality of religion through hope on satisfaction with life was significant (IE = 0.034, [LLCI = 0.003, ULCI = 0.073]). Centrality significantly fostered hope (*b* = 0.22, *t* = 3.33, *p* = 0.001), which in turn increased satisfaction with life (*b* = 0.16, *t* = 2.38, *p* = 0.018). The direct effect of the centrality of religion on satisfaction with life was also significant (DE = 0.15, t = 2.28, *p* = 0.024). The total effect was also significant (TE = 1.29, *t* = 2.83, *p* = 0.005, [LLCI = 0.394, ULCI = 2.193] (Fig. 1).

## Discussion

The present study aimed to identify possible mediators of the relationship between the centrality of religiosity and satisfaction with life in late adults (60–92 years old). We examined forgiveness (attitude and tendency to forgive, Brown, 2003) and basic hope as mediators. Theoretical concepts have allowed the assumption that religiousness is related to well-being, and the nature of this relationship is mediated by other variables (Gall et al., 2005; Park & Slattery, 2013).

The correlational analysis confirmed a positive, statistically significant correlation between the centrality of religiosity and satisfaction with life. Thus, religiousness can support the seniors’ satisfaction with life. The structure of the scale we used allows us to measure both the private, more emotional aspect of religiousness and its social facets. Both had their share in increasing satisfaction with life in the seniors. Our results are consistent with those reported by other authors who have found positive associations between religiousness and satisfaction with life (Fastame et al., 2021; Garssen et al., 2021; Krause, 2003; Park, 2005; Platsidou, 2012). Religious people can draw support from religion. Religion can foster a sense of belonging to a community and thus give social support. As well as religion can be a source of meaning for personal experiences and reinforce a sense of emotional security (Bergan & McConatha, 2001; Fastame et al., 2021; Gall et al., 2005).

Life satisfaction is a complex variable that can be explained by various factors. Research has confirmed the role of personality, health, socioeconomic status, employment situation, family status, social capital, and religion (Azizan & Mahmud, 2018; Bjørnskov et al., 2008; Pavot & Diener, 2008). The range of available resources that can determine life satisfaction narrows as the years go by. Therefore, the importance of religion may increase with age as the availability of other resources diminishes (Kongarchapatara et al., 2014; Pahlevan et al., 2021).

In seniors, a higher religiousness is associated with a higher forgiving attitude, a higher tendency to forgive, and a higher level of basic hope. This is consistent with findings reported by other researchers (Berry et al., 2005; Brown et al., 2007; Edwards et al., 2002; Park & Slattery, 2013). Our study showed that religiousness is positively associated with beliefs about forgiveness (forgiving attitude) and the situational dimension of forgiveness (tendency to forgiveness). Thus, religiousness can be a source of how people think about forgiveness (Pargament & Rye, 1998; Park, & Slattery, 2013), and it may incline individuals to engage in forgiveness-oriented activity (Krok & Zarzycka, 2021).

Our study demonstrated that the tendency to forgive was related to life satisfaction in the surveyed seniors (Brown & Phillips, 2005). This result does not align with previous research on the relationships between forgiveness and life satisfaction (Hill & Allemand, 2011; Toussaint et al., 2001). The difference can be attributed to the complex nature of the phenomenon of forgiveness and the peculiarities of how people function in the last phase of their lives. Erikson (1982) characterized the final stage of life by referring to the notion of the conflict between wisdom and despair. At this stage, people undertake a holistic reflection on life, try to rectify mistakes, reconcile with others, or reconcile conflicted parties within their circle of loved ones.

Our study confirmed the association between hope and satisfaction with life, which is consistent with other researchers (Gall et al., 2005; Garoon et al., 2016; Klein, 2016). A higher level of basic hope, defined as a perception of the world as orderly, meaningful, and valuable, can provide the elderly to use more favorable ways of adapting to the life changes and losses of old age, leading to an improved mood, well-being, and even improve physical health (Garoon et al., 2016; Klein, 2016).

Our results partially confirm the first hypothesis that forgiveness plays a mediating role in the relationship between the centrality of religiosity and satisfaction with life in seniors. Centrality was positively linked to the tendency to forgive, increasing life satisfaction. The mediating effect of the attitude toward forgiveness was not significant. Park and Slattery (2013) as well as other researchers (Lawler-Row & Elliott, 2009; Schultz et al., 2010; Worthington & Sherer, 2004) have indicated that religiosity can affect well-being by shaping positive attitudes toward forgiveness. Our study showed that what matters in seniors is not so much the attitude towards forgiveness but the willingness (tendency) to forgive. Spirituality and religion may have situational meaning, that is, meaning that is manifested in specific transactions between a person and his or her environment (Gall et al., 2005; Park & Folkman, 1997). Religious content can touch the sensibilities of seniors, leading them toward forgiveness and reconciliation with others and consequently increase well-being (Gall et al., 2005).

The centrality of religiosity had a statistically significant indirect effect on satisfaction with life through hope, supporting Hypothesis 2. Thus, the centrality of religiosity fosters hope, which in turn increases life satisfaction. Our result is in line with previous findings, which confirmed the association between religiousness and basic hope (Van Hook, 2016) and between basic hope and seniors’ well-being (Poulin & Haase, 2015). We can conclude that successful, satisfying aging can be a mutually reinforcing interplay between religiosity, forgiveness, and hope (Gall et al., 2005).

The mediation effects achieved are moderate. Thus, life satisfaction in aging is a complex process explained by numerous factors. Religion may be one of the elements contributing to seniors’ satisfaction. Centrality is a construct that does not differentiate between aspects of religiosity but captures the importance of religiousness. Distinguishing content aspects of religiosity, such as relational, belief, behavioral, and experiential elements, could shed additional light on the role of religiosity in predicting life satisfaction for seniors.

## Limitations and Future Directions

The limitation of the present study is its cross-sectional design, which does not allow for causal conclusions. Also, the respondents belong to a relatively homogeneous group representing a specific cultural context. Therefore, these results cannot be generalized to other groups and cultural contexts. In future explorations of this subject, quantitative methods should be supplemented with qualitative methods to explore subtle aspects of how older people understand life satisfaction. A shortcoming of research conducted among seniors is the need for psychological instruments adjusted to participants’ possibilities at this stage of life, especially for people over 80. Since people at this age undergo dynamic changes related to the loss of strength and increasing physical ailments, there is also a shortage of psychological tools for measuring the dynamics of changes in satisfaction with life and other variables sufficiently precisely. Undoubtedly, a longitudinal study using high-sensitivity instruments, supplemented with qualitative (conversations, interviews) and observational measures, is warranted in future research.

## Conclusion

The research results show that religiosity can contribute to higher life satisfaction among the elderly. The relationship between religiosity and life satisfaction is mediated by a tendency to forgive (situational forgiveness) and hope. Thus, being religious may be a supportive factor contributing to seniors’ life satisfaction.

## Data Availability

The data presented in this study are available on request from the corresponding author.
